# Hidden in Plain Sight: HIV and LGBTQIA+ Realities in the Middle East and North Africa Region

**DOI:** 10.1002/jia2.70122

**Published:** 2026-05-13

**Authors:** Nadia Ahmed, Elbushra Ali Mohamed Herieka, William Abou Shahla, Binta Sultan

**Affiliations:** ^1^ Department of Sexual Health and HIV Central North West London NHS Foundation Trust London UK; ^2^ Department of Sexual Health and HIV Dorset Healthcare University NHS Foundation Trust Bournemouth UK; ^3^ Department of Internal Medicine Ernst Von Bergmann Potsdam Germany; ^4^ Institute for Global Health University College London London UK

1

The Middle East and North Africa (MENA) is only one of two regions with a consistent rise in HIV incidence, increasing by 116% between 2010 and 2023 and reaching around 23,000 new cases annually [1]. Despite a relatively low general prevalence and 6% decrease in AIDS‐related deaths, local epidemics concentrate in key populations, notably men who have sex with men, among whom there has been a rapid rise in prevalence [1]. These combined trends create a prevention opportunity: services can still avert a more entrenched epidemic if safe, confidential and trusted enough for LGBTQIA+ people to use. This requires attention not only to biomedical provision, but structural, legal and social conditions shaping access to prevention, testing and care. Limited epidemiological data on HIV and LGBTQIA+ communities further hamper efforts for effective policy‐making, resource allocation and targeted interventions [2]. Conflict and displacement have also disrupted services, deepened public health vulnerabilities and made continuity of care harder to sustain. Strengthening the HIV response in MENA, therefore, means making services safer to access, while protecting and resourcing healthcare providers (HCPs), civil society groups and grassroots organizations already creating trusted routes into care.

The MENA region encompasses 22 countries, often treated as a single epidemiological category. This regional framing can help identify shared opportunities: low overall HIV prevalence, established clinical expertise, growing prevention tools and community organizations. It also highlights shared challenges: displacement, stigma, legal or policy environments making LGBTQIA+ visibility difficult [2, 3, 4]. These commonalities should not obscure diverse legal systems, histories, languages, religious traditions and public health capacities across the region. LGBTQIA+ people are not positioned in the same way across countries, with conditions shaping access to care differing accordingly. Laws vary considerably from criminalization of same‐sex conduct or gender expression to cybercrime or public order provisions potentially affecting LGBTQIA+ visibility and service access [3, 4, 5, 9, 10, 11]. Some of these legal frameworks reflect colonial‐era penal codes and imported classifications of “public morality,” while others reshaped contemporary state, religious and political contexts. Religious traditions should not be framed only as barriers; principles of compassion, dignity and non‐judgement already can support a humane and effective HIV response [4, 6]. Legal and policy reform matters, because prevention depends on trust: where people fear arrest, exposure, deportation or discrimination, they are unlikely to test, disclose, return, start or remain in care.

Stigma related to sexual orientation, gender identity and HIV status compounds across healthcare, family and communities. HCPs sometimes lack HIV knowledge and hold discriminatory attitudes, limiting culturally competent care for those at greatest HIV risk [7]. These overlapping barriers can deter LGBTQIA+, including people living with HIV (PLWH), from using healthcare systems, disclosing risk, accessing prevention and remaining in care. Fear of criminalization, incarceration or exposure also restricts advocacy and underutilization of basic prevention tools, including comprehensive sexuality education and condom provision [7, 8].

Ongoing and recent conflicts in Syria, Yemen, Libya, Iraq, Sudan, Palestine, Israel, Lebanon and Iran pose serious challenges to safe, effective HIV prevention and care. Conflict damages health infrastructure, disrupts supply chains, interrupts treatment and follow‐up, and diverts resources from public health programmes. Displacement, social network breakdown and poverty increase vulnerability, while refugees and internally displaced persons may face legal and administrative barriers to national health systems. Being LGBTQIA+ heightens exploitation, violence and exclusion risks, often without safe spaces for support. Humanitarian and HIV responses must include confidential testing, treatment continuity, prevention tools and safe referral pathways for LGBTQIA+ people.

Yet, despite these conditions, progress is visible across the MENA. Incremental legal and policy changes, improved HIV prevention‐to‐care continuum pathways and innovative outreach strategies show inclusive HIV responses are possible in complex legal and social contexts. Some countries have had progressive repealing of laws such as one that criminalized the “imitation of the opposite sex” in Kuwait (Article 198), and there has been a movement away from law enforcement by religious authorities to the secular police in Saudi Arabia [9]. Yemen's 2009 HIV anti‐discrimination law and anti‐discrimination laws in education, employment and healthcare for PLWH in Bahrain signify encouraging reforms [3]. Where LGBTQIA+ identities are negatively portrayed as foreign ideologies, activists have successfully responded by shifting to localized, context‐specific advocacy, avoiding Western models. However, restrictions persist, including against the use of the term “gender” in Iraq and Libya [10, 11], laws penalizing certain online activities in Jordan and Egypt [12], and criminalized same‐sex relations in Tunisia (Article 230).

HIV treatment access, prevention strategies, and national policy and programmes have improved in multiple contexts. Morocco reported a 35% decline in incidence between 2010 and 2023, alongside HIV prevention programmes, self‐testing and multi‐month antiretroviral therapy (ART) innovations [1]. Lebanon strengthened its response by increasing domestic funding, expanding ART and monitoring systems, while Tunisia improved HIV surveillance and prevention programmes targeting key populations [14]. Pre exposure prophylaxis (PrEP) implementation in Morocco, Lebanon, Kuwait and the United Arab Emirates, with approval pending in Saudi Arabia, reflects a willingness to adopt evidence‐based strategies to reduce HIV transmission [15]. A shift towards community‐led and other civil society organizations in healthcare for LGBTQIA+ communities continues to grow. Helem in Lebanon, ALCS in Morocco and ATL MST/SIDA in Tunisia provide targeted outreach, confidential testing and counselling services for LGBTQIA+ populations. The International Development Law Organization integrated legal support to challenge discrimination of LGBTQIA+ PLWH and improve healthcare access in Egypt, Jordan, Lebanon and Morocco.

Collective efforts addressing stigma include challenging legal barriers and conservative religious interpretations, facilitating access to counselling and clinical services. LINKAGES by Autre Regard in Djibouti provide mobile hotlines for high‐risk men, linking them to HIV prevention and treatment resources. ATL MST/SIDA and Helem utilize social media to provide anonymous HIV education, promote testing and prevention, connect users to services, and offer support and counselling. Project ECHO in Sudan uses a hub‐and‐spokes knowledge‐sharing approach through virtual clinics and innovative tele‐mentoring, reaching underserved, remote communities to reduce health disparities.

To end HIV in MENA countries, any comprehensive effort must address biomedical, behavioural, social and legal factors together. This requires policy reform, funding for trauma‐informed, non‐discriminatory HIV services, expanded access to testing and prevention, and stronger community networks, particularly in settings affected by conflict. Resourcing those already leading this work will ensure progress can continue, and HIV prevention becomes safer, more trusted and more accessible for LGBTQIA+ people across the region.

## Author Contributions

NA conceived the manuscript. EAMH, WAS and BS reviewed and provided inputs.

## Conflicts of Interest

All authors declare no conflicts of interest.

## Data Availability

Data sharing is not applicable to this article as no new data were created or analyzed in this study.
